# Resting Metabolic Rate for Diagnosing Tae-Eum Sasang Type and Unraveling the Mechanism of Type-Specific Obesity

**DOI:** 10.3390/diagnostics13040672

**Published:** 2023-02-10

**Authors:** Jieun Park, Seul Lee, Yongjae Lee, Jeongyun Lee, Han Chae

**Affiliations:** 1 Department of Sasang Constitutional Medicine, Pusan National University Korean Medicine Hospital, Yangsan 50612, Republic of Korea; 2 School of Korean Medicine, Pusan National University, Yangsan 50612, Republic of Korea; 3 Department of Sasang Constitutional Medicine, Division of Clinical Medicine 4, School of Korean Medicine, Pusan National University, Yangsan 50612, Republic of Korea

**Keywords:** Sasang typology, standardized physical measure, resting metabolic rate per weight, percent skeletal muscle, obesity

## Abstract

Increased resting metabolic rate (RMR), representing augmented energy expenditure, is a preferred physical characteristic; however, the Tae-Eum Sasang type, with a high incidence of obesity and metabolic diseases, has a higher RMR. This study scrutinized the physical characteristics of Sasang typology, a traditional Korean personalized medicine, to resolve this discrepancy, which can unravel the mechanism of Tae-Eum-type-specific obesity and improve the Tae-Eum Sasang-type diagnosis. A total of 395 healthy participants provided Sasang-type diagnosis using Sasang Constitutional Analysis Tool and physical features, including skeletal muscle mass, body fat mass, and RMR, along with those standardized using body weight. The Tae-Eum-type group showed significantly higher body weight, body mass index, body fat mass, and unstandardized RMR (kcal/day) than others, while their standardized measures of RMR per weigh (RMRw, kcal/day/kg) and percent skeletal muscle (PSM, %) were significantly lower. The logistic regression model revealed that the RMRw is pivotal for discriminating Tae-Eum type from others and explaining the developmental mechanism of Tae-Eum-type obesity. The aforementioned might provide a theoretical framework for Sasang-type diagnosis and Sasang-type-specific health promotion using bodily exercise and medical herbs.

## 1. Introduction

Resting metabolic rate (RMR) or resting energy expenditure represents the total calories used for life processes and body temperature in the resting state; it accounts for up to 70% of daily energy expenditure in sedentary people [1]. It is known to be affected by muscle mass with the influence of sex, age, ethnicity, and other factors [2]. An individual with high RMR was reported to lose weight more easily and to have a relatively slender body than others, while another individual with low RMR has a high probability of obesity [3] associated with metabolic syndrome [4,5]. Therefore, since the prevalence rate of obesity and metabolic disease was reported different following the Sasang types, there have been clinical studies on the RMR characteristics of Sasang typology, a traditional Korean personalized medicine [6,7].

**Figure 1:**
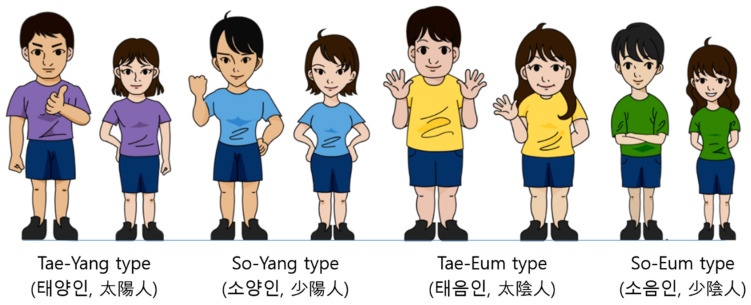
Illustrated figures of four Sasang types.

The Sasang typology divides individuals into four types of Tae-Yang, So-Yang, Tae-Eum, and So-Eum, based on physical, psychological, and clinical characteristics (Figure 1). It was established based on clinical experiences of East-Asian traditional medicine and the biopsychosocial theory of neo-Confucianism by Jema Lee in 1894. It uses the same acupuncture points and medical herbs of East-Asian traditional medicine following the Sasang-type-specific pathophysiology for higher safety and efficacy [8,9]. Three common Sasang types of So-Yang, Tae-Eum, and So-Eum were shown to be similar to the Choleric, Phlegmatic, and Melancholic humoral types of Hippocrates and Galen; the Athletic, Pyknic, and Asthenic physique types of Kretschmer; the Mesomorph, Endomorph, and Ectomorph somatotypes of Sheldon; and Pitta, Kapha, and Vata of Prakriti in Ayurveda [10].

The physical, psychological, and clinical characteristics of each Sasang type have been reported using objective measures [11,12,13,14,15]. From a psychological perspective, the So-Yang Sasang type was shown to have a high Sasang Personality Questionnaire (SPQ), Novelty-Seeking Temperament and Character Inventory (TCI), and Extraversion NEO-Personality Index scores, and lower Harm-Avoidance TCI score, while the So-Eum-type group had contrasting features [10,13,15,16,17].

From a physical perspective, the Tae-Eum and So-Eum Sasang types were reported to have contrasting physical characteristics; the Tae-Eum type was known to have higher body weight, height, body mass index (BMI) [12,18], and ponderal index (PI) [6] compared to the So-Eum type. The Tae-Eum type was reported to have a higher incidence of obesity [6,13,19], hypertension [20,21], diabetes [22,23], cardiovascular disease [24], and metabolic diseases [14,24,25], and the So-Eum-type group was on the contrary.

However, contrary to the expectations, the RMR of the Tae-Eum-type group was reported to be higher than that of other groups, which could misguide that the Tae-Eum type has a relatively slender body from augmented energy expenditure and lowered incidence of obesity and metabolic diseases [6,7]. Previous studies have suggested hypotheses to explain this discrepancy; one suggested that the difference between calculated and directly measured RMRs might have caused the confusion [6], and another proposed that the uneven mitochondrial density between Sasang types at the cellular level could explain the discrepancy [7]. Nevertheless, rational explanations and/or tangible evidence have yet to be presented to date [13].

To address this issue, the current study examined the physical characteristics of each Sasang type using the body composition of skeletal muscle mass and body fat mass, which are related to the regulation of energy metabolism [2,26]. Furthermore, unstandardized and standardized (with body weight) body compositions and RMR of each Sasang type were compared to unravel the Sasang-type-specific resting energy metabolism leading to obesity [27]. Additionally, both measured RMR [7] and calculated RMR [6] were also obtained to examine the possible clinical dissimilarities between them in the Sasang typology.

The physical characteristics that could unravel the Sasang-type-specific energy metabolism and improve the Sasang-type diagnosis were assessed, and the physiological tendencies and pathological characteristics of each Sasang type were scrutinized. Thus, the differentiation of Sasang types using objective measures along with Sasang-type-specific interventions with bodily exercise and medical herbs will be provided.

## 2. Methods and Materials

### 2.1. Procedures and Participants

A total of 395 healthy participants were recruited using advertisement with a leaflet, banner, and poster and were asked to visit once for physical measures and Sasang-type diagnosis. Those who stated they had cardiovascular disease, cerebrovascular disease, malignant neoplasm, mental disease, arthritis, thyroid disease, or pregnancy were excluded from the study.

The trained clinical research coordinator arranged the date and time of the visit with the participants and completed the physical measures. Demographic features of age and sex, physical characteristics of height, weight, body composition (skeletal muscle mass (SMM), body fat mass (BFM)), and RMR (RMR_meas) along with diagnosed Sasang types were acquired, and the physical features of BMI, PI, and RMR (RMR_calc) were calculated.

This study was performed at the Pusan National University Korean Medicine Hospital between September 2020 and December 2021. This study was approved by the Institutional Review Board of Pusan National University Korean Medicine Hospital (PNUKHIRB-202005, PNUKHIRB-2021002), and all the participants provided informed written consent for this study after they were apprized about the research purpose, measures, potential benefits, and risks in advance.

### 2.2. Physical Characteristics

The physical characteristics, including height (cm) and weight (kg), were measured using a digital stadiometer (BSM370, InBody Co., Seoul, Republic of Korea). SMM (kg) and BFM (kg) were measured using a bioelectric impedance analyzer (BWA 2.0, InBody Co., Seoul, Republic of Korea), which estimates body composition [18]. The participants were asked to lay on the bed while maintaining the trunk and limbs separated, with clamp-type sensors on both wrists and ankles; their body composition was measured after resting for 5 min.

The RMR (RMR_meas, kcal/day) was acquired using Quark RMR (COSMED Srl., Albano Laziale, Italy), which examines energy expenditure by measuring the actual respiratory quotient divided by oxygen consumption (VO_2_) and carbon dioxide production (VCO_2_). The participants were asked to sit on a chair for 15 min with a mask on their faces.

Physical measures, including BMI (kg/m^2^), PI (kg/m^3^), and RMR (RMR_calc, kcal/day), were calculated using the measured physical features. The BMI of obesity measure was calculated as body weight divided by the square of the height in meters, and the PI, also known as a corpulence index or Rohrer’s index, showing physical development, was calculated as body weight divided by three times the height. The RMR (RMR_calc, kcal/day) was calculated using the World Health Organization formula (male; 15.4 W − 0.27 H + 717 (age < 31), 11.3 W + 0.16 H + 901 (30 < age < 61), 8.8 W + 11.28 H − 1071 (60 < age)—female; 13.3 W + 3.34 H + 35 (age < 31), 8.7 W − 0.25 H + 865 (30 < age < 61), 9.2 W + 6.37 H − 302 (60 < age)) [12] with acquired age, gender, height, and weight of the participants. The standardized physical measures of percent skeletal muscle (PSM, %), percent body fat (PBF, %), RMRw_meas (kcal/day/kg), and RMRw_calc (kcal/day/kg) were acquired by dividing SMM, BFM, and RMR_meas and RMR_calc by body weight, respectively.

### 2.3. Sasang-Type Classification

The Sasang type of the participants was diagnosed using the Sasang Constitutional Analysis Tool, which collects characteristics of front and side faces, body shape with circumferences, voice recording, biopsychological traits, and pathophysiological symptoms for digestion, defecation, urination, and others, and provides a percentile index as a probability value for the four Sasang types [28,29,30]. The Sasang type with the highest probability value was suggested as the diagnosed Sasang type of the participants. The correctly predicted percentage when compared to the differential diagnosis of certified clinical specialists of Sasang typology was reported to be 71.0% for males and 66.7% for females [29]. The current study excluded the Tae-Yang-type group for the analysis since their prevalence was suggested as less than one percent by Jema Lee, and the rarity might cause misunderstanding on their physical characteristics without a sufficiently big sample size.

### 2.4. Statistical Analysis

The demographic characteristics of the participants were described using descriptive statistics. The differences in physical characteristics between males and females were examined using Student’s *t*-test. The correlation between physical measures was tested using Pearson’s correlation, and the correlation coefficient was presented. Differences in physical measures among the Sasang types were examined using analysis of covariance considering sex and post hoc analysis with Bonferroni correction.

Logistic regression analysis with RMRw, PSM, PI, BMI, and sex was used to predict Tae-Eum- and non-Tae-Eum-type groups and to compare the diagnostic significance of predictors. The model fit was examined using the chi-squared test and Nagelkerke’s R², and the percentage of correctly classified was also acquired.

The data are presented as frequencies or means and standard deviations, and estimated values considering sex are presented as means and standard errors. All analyses were performed using IBM SPSS Statistics 26.0 (IBM, Armonk, NY, USA), and *p*-values of 0.05, 0.01, and 0.001 were used for the test of significance.

## 3. Results

### 3.1. Demographic Features

There were 393 participants (105 males and 288 females), excluding two Tae-Yang Sasang types, who showed a significant (*p* = 0.003) difference in age. Height, weight, BMI, PI, SMM, BFM, RMR_calc, and RMR_meas were significantly higher in males than in females (Table 1). The distribution of Sasang-type groups was significantly (*p* < 0.001) different between males and females.

### 3.2. Correlation between Physical Characteristics

The results of the correlation analysis between unstandardized and standardized physical measures are shown in Table 2. The RMR_calc was significantly (*p* < 0.001) correlated with BMI (r = 0.651), PI (r = 0.381), SMM (r = 0.943), BFM (r = 0.428), PSM (r = 0.41), and PBF (r = −0.228). There was a significant positive correlation between RMR_calc and RMR_meas (r = 0.716, *p* < 0.001) and RMRw_calc and RMRw_meas (r = 0.391, *p* < 0.001), which indicates that previous studies with the measured and calculated RMR should be considered as with the same reliability, and both RMRw_calc and RMRw_meas might be used for the clinical diagnosis of Sasang typology.

Interestingly, there was a positive correlation (r = 0.283, *p* < 0.001) between SMM and BFM; however, a negative correlation (r = −0.974, *p* < 0.001) between PSM (standardized SMM with body weight) and PBF (standardized BFM with body weight), which clearly showed that the standardization with body weight made the correlation between body fat and skeletal muscle volume reverse.

### 3.3. Differences in Physical Characteristics between Sasang Types

The prevalence of the Sasang type was 117 (25 males and 92 females), 196 (70 and 126), and 78 (10 and 68) for the So-Yang, Tae-Eum, and So-Eum types, respectively (Table 1), and there were significant (χ^2^ = 17.78, df = 2, *p* < 0.001) differences between males and females.

There were significant (*p* < 0.001) differences in the unstandardized and standardized physical measures among the Sasang types, as shown in Table 3. The Tae-Eum type showed significantly higher height, weight, BMI, PI, SMM, BFM, RMR_meas, RMR_calc, and PBF than the So-Yang and So-Eum, while the Tae-Eum type had significantly lower PSM, RMRw_meas, and RMRw_calc than the other Sasang types.

### 3.4. Classification of Tae-Eum- and Non-Tae-Eum-Type Groups

The results of logistic regression with sex, RMRw_calc, PSM, PI, and BMI were found to have an acceptable model fit measured with χ^2^ (211.717 for model 1 and 209.541 for model 2) and Nagelkerke R^2^ (0.564 for model 1 and 0.551 for model 2) (Table 4 and Table 5). The regression model 1 revealed the RMRw_calc as a significant predictor, which shows that the RMRw_calc is more pivotal than the BMI, PI, and PSM for classifying Tae-Eum Sasang type from others (Table 4). Additionally, the correctly predicted percentage of regression model 2 (80.7%) using sex and RMRw_calc is similar to that of regression model 1 (81.3%) using five predictors of sex, RMRw_calc, BMI, PI, and PSM (Table 5).

## 4. Discussion

This study examined the physical features and body composition of the Sasang typology using unstandardized and standardized physical measures and scrutinized the Sasang-type-specific mechanism of obesity in the Tae-Eum type [6,7,31]. The Tae-Eum-type group showed significantly higher body weight, BFM (kg), PBF (%), and unstandardized RMR (kcal/day) than others, while their standardized measures of RMRw (RMR per weight, kcal/day/kg), and PSM (%) were significantly lower (Table 3). The logistic regression models for discriminating Tae-Eum type from others showed the calculated RMRw as a pivotal clinical predictor (Table 4 and Table 5).

The standardized physical measures of PSM (%) and RMRw (kcal/day/kg) were revealed to explain the physiological predisposition of the Tae-Eum Sasang type, and unstandardized measures of weight, BMI, and BFM were disclosed as sequential pathologic features of them [2]. The lowered volume of skeletal muscle per weight (PSM) of the Tae-Eum type was found to reduce their energy expenditure per weight (RMRw), which leads to obesity and metabolic diseases [3]. However, since their total weight is substantially big enough, the RMR (weight × RMRw) representing total calories used by the Tae-Eum type was presented to be higher than others.

The current study provided considerable understanding of the pathophysiological characteristics of the Sasang typology from the following perspectives.

First, this study showed that RMRw and PSM are fundamental physical features of the Sasang typology that can be used for accurate Sasang-type diagnosis. The Tae-Eum group was shown to have higher muscle mass, lower PSM, and lowered RMRw, while the So-Eum group had lower muscle mass, higher PSM, and elevated RMRw, which were not reported previously (Table 3). In addition, the regression analysis (Table 4 and Table 5) showed that the clinical importance of RMRw for diagnosing Sasang types is higher than that of PI and BMI reported in previous studies [6,12,18], and the regression model with RMRw can classify Tae-Eum type from others with a diagnostic accuracy of 80.7%.

The distinctive physical predisposition of RMRw, PSM, PI, and BMI [6,10,12,13,32,33] might be clinically useful for objective and efficient Sasang-type diagnosis [32,34] when psychological characteristics of Novelty-Seeking and Harm-Avoidance of TCI and SPQ are considered simultaneously (Figure 1). The combination of RMRw and SPQ as physical and psychological features for clinical diagnosis might exceed 82% of diagnostic accuracy, which was reported with BMI and SPQ in previous studies [17,34,35].

Second, the results provide a theoretical framework for the tailored prevention and management of obesity and metabolic diseases rather than the stereotyped interventions of calorie restriction and appetite suppression in everyday life.

The high waist–hip ratio, BMI, PI, BFM, and PBF might be just consequential characteristics of the Tae-Eum-type group, while lowered RMRw from low PSM could be a pivotal developmental mechanism of the Tae-Eum-type-specific physical predisposition [2,26]. The prevailing obesity management strategy has regarded the reduction in fat mass as a major concern, whereas the Tae-Eum-type-specific approach recommends for management and interventions to focus on the increase in muscle mass and energy expenditure.

For example, the Tae-Eum-type-specific medical herbs were shown to modulate metabolic regulation [8]. Ephedra Sinica showed the augmentation of energy expenditure [36,37], and Platycodon Grandiflorus root increased energy expenditure and thermogenic gene expression [38]. A physical exercise scheme of high weight with low-repetition exercise and short-term anaerobic exercise for increasing muscle mass might be an optimal exercise plan for Tae-Eum-type obesity. A clinical study attesting the effectiveness of obesity management using Tae-Eum-type-specific medical herbs and acupuncture treatment is required.

Third, the current study resolved controversies regarding the high RMR (kcal/day) score of the Tae-Eum type with the use of standardized physical measures. The high body weight (kg) of the Tae-Eum-type group made them have both high RMR (kcal/day) and low RMRw (kcal/day/kg) simultaneously. In other words, the total energy expenditure of the Tae-Eum-type group is significantly higher than other groups even with their substantially smaller energy expenditure per weight because they have enough large body weight to reverse the correlation, which is the same with body fat mass and skeletal muscle volume (Table 2).

One study suspected that the disparity between calculated [6] and measured [7] RMRs might cause a misunderstanding of the physical characteristics of the Tae-Eum-type group. Nevertheless, both RMR and RMRw in the current study shared similar clinical characteristics; calculated and measured RMRs (r = 0.716) and calculated and measured RMRws (r = 0.531) showed significant (*p* < 0.001) correlations, and the three Sasang-type groups showed significant (*p* < 0.001) differences in both calculated and measured RMRs and RMRws (Table 2 and Table 3). Another previous study [7] suggested a mitochondria hypothesis that the Tae-Eum type has relatively higher cellular activity or mitochondrial density than other Sasang types. However, genetic studies on Sasang typology could not reveal the influence of sex that might be related to the maternal inheritance of the mitochondria [11].

This study still has some limitations concerning the generalization of the findings. First, the mean age of the 393 participants was around mid-forties, and further studies using diverse age groups, including adolescent, young, and aged participants, are required. Further studies are also needed to attest whether similar physical characteristics of each Sasang-type group are retained, even in patients with hypertension, diabetes, and cardiovascular disease.

Second, the BMI of the Tae-Eum-type group was shown to be in the range of overweight in the current study. Though overweight is the typical physical feature of Tae-Eum Sasang-type groups [12], selection bias still may exist during the recruitment. The high RMRw and PSM of the Tae-Eum-type group should be reexamined using a cohort or community group.

Third, the clinical usefulness of RMRw and PSM in Sasang typology should be replicated using other Sasang-type classification methods [39,40] along with the diagnosis of certified clinical specialists for generalization purposes [34]. The SMM and BFM were estimated using bioimpedance analysis and the RMR using the WHO formula; this should be attested using alternative methods to improve the level of evidence [6].

Fourth, the physical characteristics of RMRs should be examined in relation to other clinical characteristics, including psychological features of SPQ [10,16] and TCI [15], clinical symptoms of Digestive Function Inventory [33,35], Sasang Urination Defecation Inventory [41], Sasang-type-specific pathophysiological symptoms [13], and everyday lifestyles [42] from the perspective of Sasang-type diagnoses.

In conclusion, the pathophysiological characteristics of RMRw and its diagnostic usefulness in Sasang typology were presented for the first time. This study might be useful for establishing a clinical guideline for objective Sasang-type diagnoses and Sasang-type-specific health promotions and tailored medical interventions on obesity and metabolic disease with high safety and efficacy.

## Figures and Tables

**Table 1:** Demographic features of the current study.

	Male	Female	Statistics
N (%)	107 (27.1 %)	288 (72.9 %)	
Age (years)	45.89 ± 11.73	42.14 ± 10.46	*p* = 0.003
Height (cm)	173.36 ± 6.32	160.16 ± 5.32	*p* < 0.001
Weight (Kg)	77.59 ± 10.48	58.06 ± 8.62	*p* < 0.001
Sasang Type			*p* < 0.001
Tae-Yang	2 (1.9%)	0 (0%)	
So-Yang	25 (23.4 %)	94 (32.6 %)	
Tae-Eum	70 (65.4 %)	126 (43.8 %)	
So-Eum	10 (9.3 %)	68 (23.6 %)	
BMI (Kg/m^2^)	25.77 ± 2.81	22.65 ± 3.32	*p* < 0.001
PI (Kg/m^3^)	14.88 ± 1.69	14.17 ± 2.22	*p* < 0.001
SMM (Kg)	31.48 ± 4.02	20.29 ± 2.46	*p* < 0.001
BFM (Kg)	21.32 ± 6.06	19.92 ± 5.87	*p* = 0.044
RMR_calc (kcal/day)	1762.80 ± 181.60	1323.39 ± 90.74	*p* < 0.001
RMR_meas (kcal/day)	1823.75 ± 336.35	1394.9 ± 227.05	*p* < 0.001

BMI, Body Mass Index; PI, Ponderal Index; SMM, Skeletal Muscle Mass; BFM, Body Fat Mass; RMR, Resting Metabolic Rate; RMR_calc, RMR calculated using the WHO formula; RMR_meas, measured RMR.

**Table 2:** Correlation coefficient between unstandardized and standardized physical measures.

	1	2	3	4	5	6	7	8	9	10
1. Age (years)	1									
2. BMI (Kg/m^2^)	0.217 ***									
3. PI (Kg/m^3^)	0.268 ***	0.940 ***								
4. SMM (Kg)	0.001	0.602 ***	0.319 ***							
5. BFM (Kg)	0.176 ***	0.842 ***	0.804 ***	0.283 ***						
6. RMR_calc (kcal/day)	−0.045	0.651 ***	0.381 ***	0.943 ***	0.428 ***					
7. RMR_meas (kcal/day)	−0.147 **	0.481 ***	0.269 ***	0.757 ***	0.250 ***	0.716 ***				
8. PSM (%)	−0.169 ***	−0.198 ***	−0.396 ***	0.584 ***	−0.596 ***	0.412 ***	0.418 ***			
9. PBF (%)	0.181 ***	0.384 ***	0.543 ***	−0.408 ***	0.742 ***	−0.228 ***	−0.284 ***	−0.974 ***		
10. RMRw_calc (kcal/day/kg)	−0.006	−0.758 ***	−0.702 ***	−0.341 ***	−0.826 ***	−0.368 ***	−0.327 ***	0.410 ***	−0.555 ***	
11. RMRw_meas (kcal/day/kg)	−0.322 ***	−0.422 ***	−0.423 ***	−0.102 *	−0.509 ***	−0.183 ***	0.445 ***	0.362 ***	−0.437 ***	0.391 ***

*, *p* < 0.05; **, *p* < 0.01; ***, *p* < 0.001. Bold represents coefficient larger than 0.4. BMI, Body Mass Index; PI, Ponderal Index; SMM, Skeletal Muscle Mass; BFM, Body Fat Mass; RMR, Resting Metabolic Rate; RMR_calc, RMR calculated using WHO formula; RMR_meas, measured RMR; PSM, Percent Skeletal Muscle; PBF, Percent Body Fat; RMRw, Resting Metabolic Rate per weight; RMRw_calc, RMRw calculated using WHO formula; RMRw_meas, measured RMRw.

**Table 3:** Estimated physical characteristics of each Sasang type using analysis of covariance.

	So-Yang	Tae-Eum	So-Eum	Statistics
Physical measure				
Height (cm)	162.93 ± 0.51	164.55 ± 0.4	162.69 ± 0.64	F = 147.77, *p* < 0.001 (TE > SY = SE)
Weight (Kg)	59.04 ± 0.67	68.90 ± 0.52	55.65 ± 0.83	F = 263.70, *p* < 0.001 (TE > SY > SE)
BMI (Kg/m^2^)	22.14 ± 0.24	25.36 ± 0.19	20.83 ± 0.29	F = 107.07, *p* < 0.001 (TE > SY > SE)
PI (Kg/m^3^)	13.60 ± 0.16	15.45 ± 0.13	12.79 ± 0.20	F = 53.61, *p* < 0.001 (TE > SY > SE)
SMM (Kg)	22.36 ± 0.25	24.66 ± 0.19	21.34 ± 0.31	F = 491.37, *p* < 0.001 (TE > SY > SE)
BFM (Kg)	17.75 ± 0.46	23.54 ± 0.36	15.96 ± 0.58	F = 56.12, *p* < 0.001 (TE > SY > SE)
RMR_calc (kcal/day)	1416.07 ± 6.60	1509.67 ± 5.18	1387.73 ± 8.21	F = 103.54, *p* < 0.001 (TE > SY > SE)
RMR_meas (kcal/day)	1447.60 ± 22.92	1591.15 ± 17.92	1399.33 ± 28.41	F = 90.41, *p* < 0.001 (TE > SY = SE)
Standardized physical measure				
PSM (%)	37.53 ± 0.27	35.67 ± 0.21	38.08 ± 0.34	F = 101.75, *p* < 0.001 (TE < SY = SE)
PBF (%)	30.27 ± 0.47	34.30 ± 0.37	28.83 ± 0.59	F = 70.47, *p* < 0.001 (TE > SY = SE)
RMRw_calc (kcal/day/kg)	24.09 ± 0.14	22.05 ± 0.11	25.16 ± 0.17	F = 136.92, *p* < 0.001 (TE < SY < SE)
RMRw_meas (kcal/day/kg)	24.57 ± 0.35	23.26 ± 0.27	25.44 ± 0.43	F = 7.59, *p* < 0.001 (TE < SY = SE)

BMI, Body Mass Index; PI, Ponderal Index; SMM, Skeletal Muscle Mass; BFM, Body Fat Mass; RMR, Resting Metabolic Rate; RMR_calc, RMR calculated using WHO formula; RMR_meas, measured RMR; PSM, Percent Skeletal Muscle; PBF, Percent Body Fat; RMRw, Resting Metabolic Rate per weight; RMRw_calc, RMRw calculated using the WHO formula; RMRw_meas, measured RMRw.

**Table 4:** Logistic regression analysis for the classification of Tae-Eum- and non-Tae-Eum-type groups.

	Predictors	B	SE	Wald	*p*-Value	Exp (B)	95% CI for Exp (B)
Model 1	Sex	−4.822	2.492	3.744	0.053	0.008	[0, 1.064]
	RMRw_calc **	1.712	0.584	8.591	0.003	5.542	[1.764, 17.415]
	PSM	0.010	0.058	0.031	0.860	1.010	[0.902, 1.131]
	PI	−1.659	1.052	2.485	0.115	0.190	[0.024, 1.498]
	BMI	1.281	0.959	1.785	0.182	3.600	[0.550, 23.569]
Model 2	Sex ***	−1.869	0.334	31.259	<0.001	0.992	[0.080, 0.297]
	RMRw_calc ***	1.141	0.117	94.952	<0.001	0.178	[2.489, 3.939]

**, *p* < 0.01; ***, *p* < 0.001. RMRw_calc, Resting Metabolic Rate per weight calculated using WHO formula; PSM, Percent Skeletal Muscle; PI, Ponderal Index; BMI, Body Mass Index.

**Table 5:** Classification table and model summary of logistic regression analysis for discriminating Tae-Eum- and non-Tae-Eum-type groups.

			Predicted		PCP (%)	Χ^2^	Nagelkerke R^2^
			Tae-Eum	Non-Tae-Eum			
Model 1	Observed	Tae-Eum	161	32	83.4		
		Non-Tae-Eum	40	152	79.2		
	Overall				81.3	Χ^2^ = 211.717, *p* < 0.001	0.564
Model 2	Observed	Tae-Eum	159	37	81.1		
		Non-Tae-Eum	39	158	80.2		
	Overall				80.7	Χ^2^ = 209.541, *p* < 0.001	0.551

PCP, Percentage correctly predicted.

## Data Availability

The data presented in this study are available on request from the corresponding author. The data are not publicly available due to the lack of consent from the participants.
